# Treatment of scaphoid fractures and pseudarthroses with the human allogeneic cortical bone screw. A multicentric retrospective study

**DOI:** 10.1186/s10195-023-00686-7

**Published:** 2023-02-10

**Authors:** Simon Sailer, Simon Lechner, Andreas Floßmann, Michael Wanzel, Kerstin Habeler, Christian Krasny, Gudrun H. Borchert

**Affiliations:** 1 Bezirkskrankenhaus Schwaz Betriebsgesellschaft mbH, Swarovskistraße 1-3, 6130 Schwaz, Austria; 2 Universitätsklinikum St. Pölten, Matthias-Corvinus Straße 45, 3100 St. Pölten, Austria; 3 Unternehmung Wiener Gesundheitsverbund Klinik Ottakring, Montleartstraße 37, 1160 Wien, Austria; 4 Lehrkrankenhaus Wiener Neustadt, Erwin-Schrödinger-Straße 4, 2700 Wiener Neustadt, Austria; 5 Orthopädisches Spital Speising Wien, Speisinger Straße 109, 1130 Wien, Austria; 6 Dr. Borchert Medical Information Management, Egelsbacher Str. 39E, 63225 Langen, Germany

**Keywords:** Human allogeneic cortical bone screw, Shark Screw®, Scaphoid fracture, Pseudarthroses, Delayed union, Proximal pole, Union rate, Multicenter retrospective study

## Abstract

**Background:**

Allograft bone screws are rarely described for the fixation of the scaphoid. When fresh fractures are treated, metal screws are mainly used; when pseudarthrosis is the indication, plates in combination with vascularized or non-vascularized bone grafts are mainly used. The necessity of metallic screw removal is under debate, but it is mandatory for plates because of movement restrictions due to the plate. The use of biomaterials in scaphoid fracture fixation was described as leading to union rates of between 64 and 100%. Brcic showed the incorporation of an allogeneic cortical bone screw at 10 weeks postoperative, along with revascularization and stable osteosynthesis with primary bone healing, without any signs of immunological rejection. The purpose of this retrospective study was to explore the results obtained using an allogenic cortical bone screw (Shark Screw®) in patients with fresh scaphoid fracture fixation and pseudarthroses with respect to union rates and time to union.

**Patients and methods:**

We retrospectively analyzed 75 patients: 31 with fresh fractures and 44 pseudarthrosis patients. The Shark Screw® was used for the fixation of the scaphoid in the fresh-fracture and pseudarthrosis patients. We evaluated the union rate, complication rate and time to union.

**Results:**

Using the human allogeneic cortical bone screw for scaphoid fracture fixation led to a high union rate (94–96%). There were two nonunions in the fresh fracture group and two nonunions in the pseudarthrosis group. The complication rate was 1.3% (1 patient). Median time to union was 16, 18 and 29 weeks for the fresh-fracture, pseudarthrosis and delayed-union patients, respectively. The treatment of fresh scaphoid fractures and pseudarthroses showed similar union rates to those described in the literature, uses a shorter and less invasive surgical method with no need for hardware removal, and has a low complication rate.

**Conclusion:**

Using the human allogenic cortical bone screw (Shark Screw®) led to similar union rates in fresh fractures—but better union rates in pseudarthrosis patients—compared to those presented in the literature for other scaphoid fracture fixation techniques, and it enabled a short and low-invasive procedure without any donor site morbidity and without the necessity to remove the hardware in a second surgery. The pseudarthrosis patient group showed a particularly strong benefit from this new procedure. The physiological bone metabolism remodels the cortical bone screw without scars.

*Level of evidence:* III: retrospective cohort study, therapeutic investigation of a treatment.

## Introduction

**Fig. 1:**
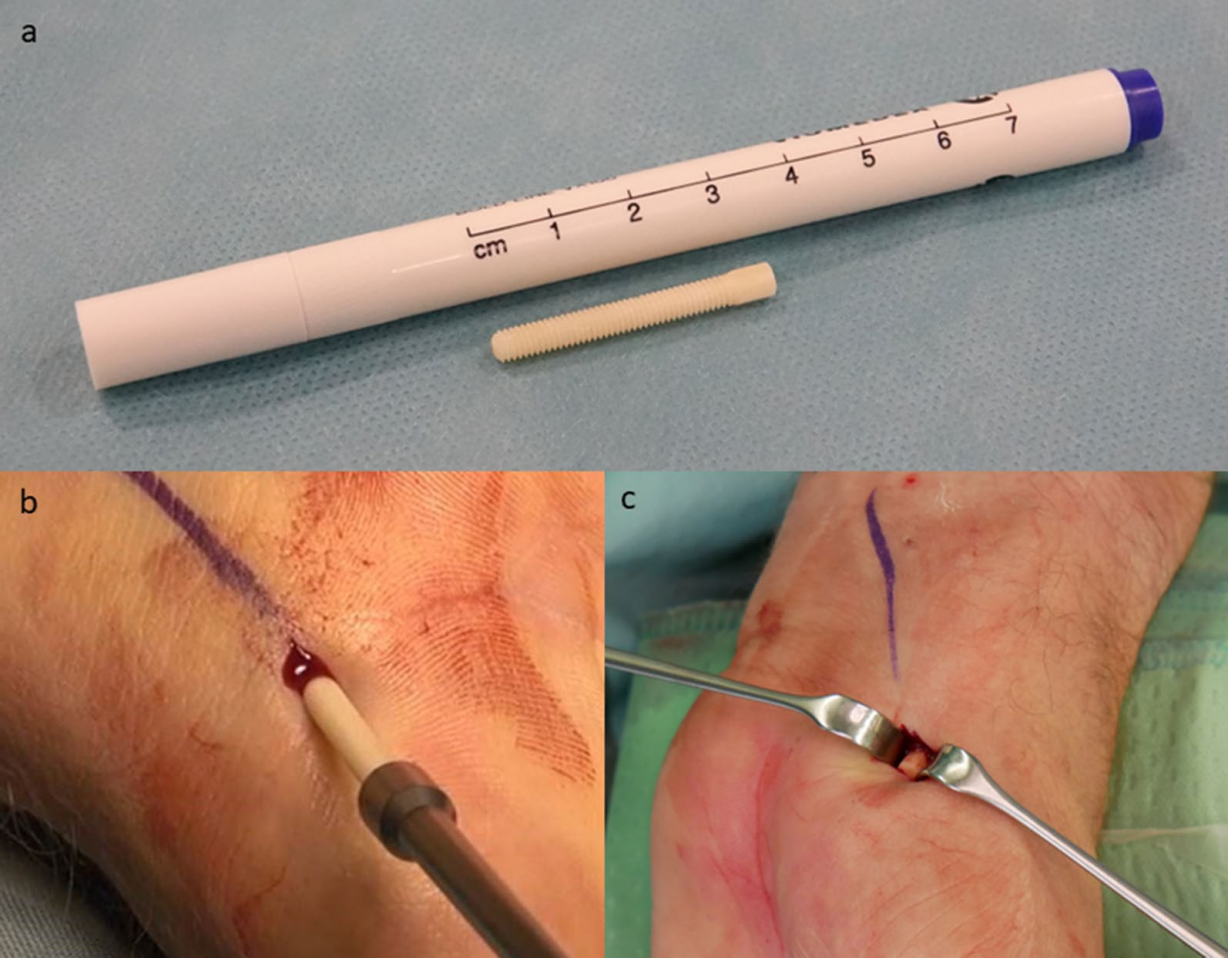
Bone screw and surgical procedure.** A** Example of the cortical bone screw. **B** The Shark Screw® "cut" is screwed in, without much resistance, with a hexagonal screwdriver. **C** The skin incision was pulled proximally with a narrow-wound hook and, at the same time, the wrist was bent so that the protruding material of the cortical bone screw could be sawn off just above the bone surface in a subsequent step

Allograft use for scaphoid reconstruction was described for the first time by Carter et al. [1] using Herbert screws (metal screws) for fixation. The necessity of metallic screw removal is under debate [2]. Wang et al. [3] described that 89% of patients insisted on screw removal because of their cultural belief. Others report that screw removal was required owing to pain at the scaphotrapezial joint [4], and removal rates of 8–14% have been reported [5]. The use of biomaterials in scaphoid fracture fixation was described [2] as leading to union rates of between 64 and 100%, but no human allogeneic cortical bone screws were described in that review. At the same time, Manako et al. [6] used autologous bone screws with good integration over time [6]. But high donor site morbidity and a long operation time were also associated with this technique [6]. Human allogeneic cortical bone screws (Shark Screw®, Fig. 1) were shown to be a substantial alternative to titanium screws due to their biomechanical properties [7]. Brcic [8] showed the incorporation of an allogeneic cortical bone screw at 10 weeks postoperative, along with revascularization and stable osteosynthesis with primary bone healing, without any sign of immunological rejection. Pastl et al. [9] described the first results of using the allogeneic cortical bone screw in hand and foot surgery. Several months after surgery, the human allogeneic cortical bone screw was no longer distinguishable from the host bone [9]. In their very recent study, Amann and Bock [10] described that, in all patients, the human allogeneic cortical bone screw showed good radiological integration into host bone after 12 months when used for tarsometatarsal II/ + III joint arthrodesis. Similar results were described by Huber et al. [11] when using the Shark Screw® for a modified chevron osteotomy, and by Hanslik-Schnabel et al. [12] when using it for the correction of hallux rigidus with and without deformities.

Scaphoid fractures account for 50–90% of all carpal fractures in young active patients [13–15]. Approximately 10% [13, 16–18] of all scaphoid fractures progress to nonunion, dependent on the fracture location, fracture displacement, instability and time to treatment [17]. Delayed diagnosis and consequently delayed treatment is an avoidable complication and often leads to nonunion and additional surgeries [15, 19]. The accepted standard treatment of scaphoid nonunion is non-vascularized grafting with internal fixation [19, 20]. The main goal of scaphoid reconstruction is to obtain union and a normal shape and length of the scaphoid [20]. Even in non-displaced acute fractures, the nonunion rate is 4–10% [13, 14]. Different methods of treatment are compared by Pinder et al. [17], who found no superiority of one over the other. Union rates of scaphoid fracture are reported to be 97.3%, 92.6% and 82% for middle, distal and proximal fractures, respectively [20]. Union rates in the presence of a proximal scaphoid nonunion and avascular necrosis are only 12–50% [19, 21]. Proximal scaphoid pseudarthroses show even lower rates of union [22], a longer healing time [23], and are much more difficult to treat [24]. Times from injury to surgery of greater than 31 days were associated with nonunion [25]. A nonunion rate of 19% in the waist region when reconstruction was delayed was reported [26]. Delayed union or nonunion of the scaphoid leads to persistent pain, reduced mobility, degenerative changes in carpal bones [18] and wrist instability [19]. It is a common opinion in the literature that vascularized grafts are preferred over non-vascularized grafts for the treatment of pseudarthroses [18, 27, 28], but the superiority of vascularized grafts is still unproven [23, 24, 29]. When this technique is used, union rates are still low (63–89%) [28–30], and donor site morbidity is a crucial factor. Especially in pseudarthroses with a D2 or higher classification, union rates are low [31]. The iliac crest, the medial and lateral femoral condyles and the distal radius are the main sources of grafts [17, 19, 24, 29, 32]. Iliac crest use was associated with a 9% complication rate [17], but complications are poorly reported [17]. The human allogeneic cortical bone screw is an allograft. No autograft is mandatory for scaphoid fixation. Using the human allogeneic cortical bone screw circumvents all the issues with donor site morbidity.

The purpose of this retrospective study was to examine the first results of using the human allogenic cortical bone screw for fresh scaphoid fracture fixation and pseudarthroses with respect to union rates and time to union. Complication rates will be a second outcome. The hypothesis is that union rates when using the Shark Screw® are at least similar to union rates described in the literature for other scaphoid fracture fixation techniques.

## Methods

We received approval from the institutional review boards (1468/2020, GS1EK-4/693-2020 and EK20-355-VK) before the beginning of the study. Due to ethical concerns, we did not use a control group who received a metal screw instead of the bone screw. Because of the retrospective nature of the study, the need for an informed consent was waved. No involvement of patients in the data collection and data treatment was necessary. All post-surgery visits were finished before the start of the study. The study was performed in accordance with the principles of the Declaration of Helsinki.

**Fig. 2:**
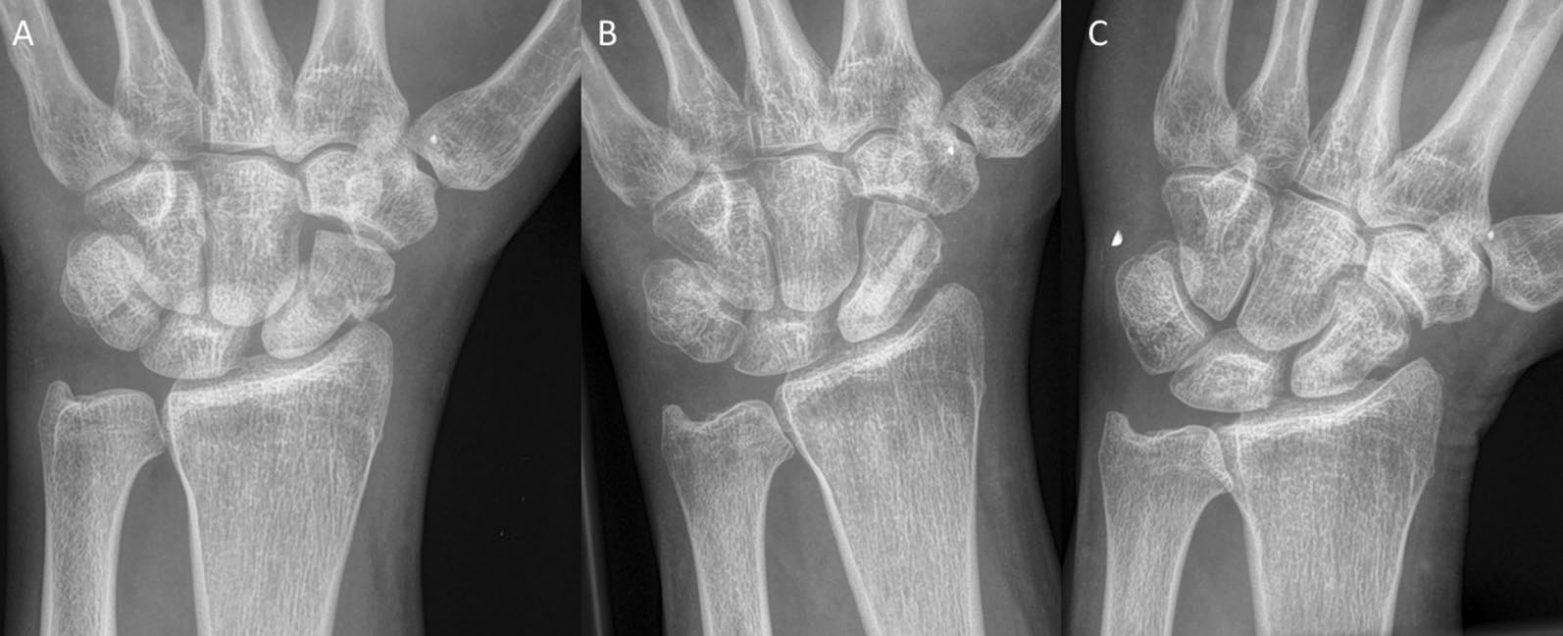
Fresh scaphoid fracture in the middle third. **A** Pre-surgery. **B** 8 weeks post-surgery; **C** 16 months post-surgery

**Fig. 3:**
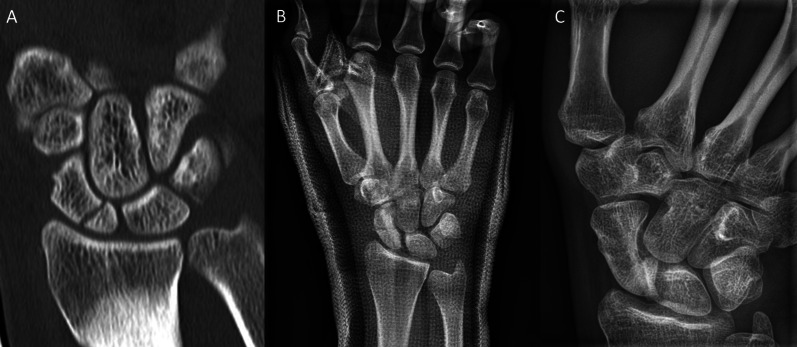
Fresh scaphoid fracture in the proximal pole. **A** Pre-surgery. **B** 1 day post-surgery. **C** 12 months post-surgery

**Fig. 4:**
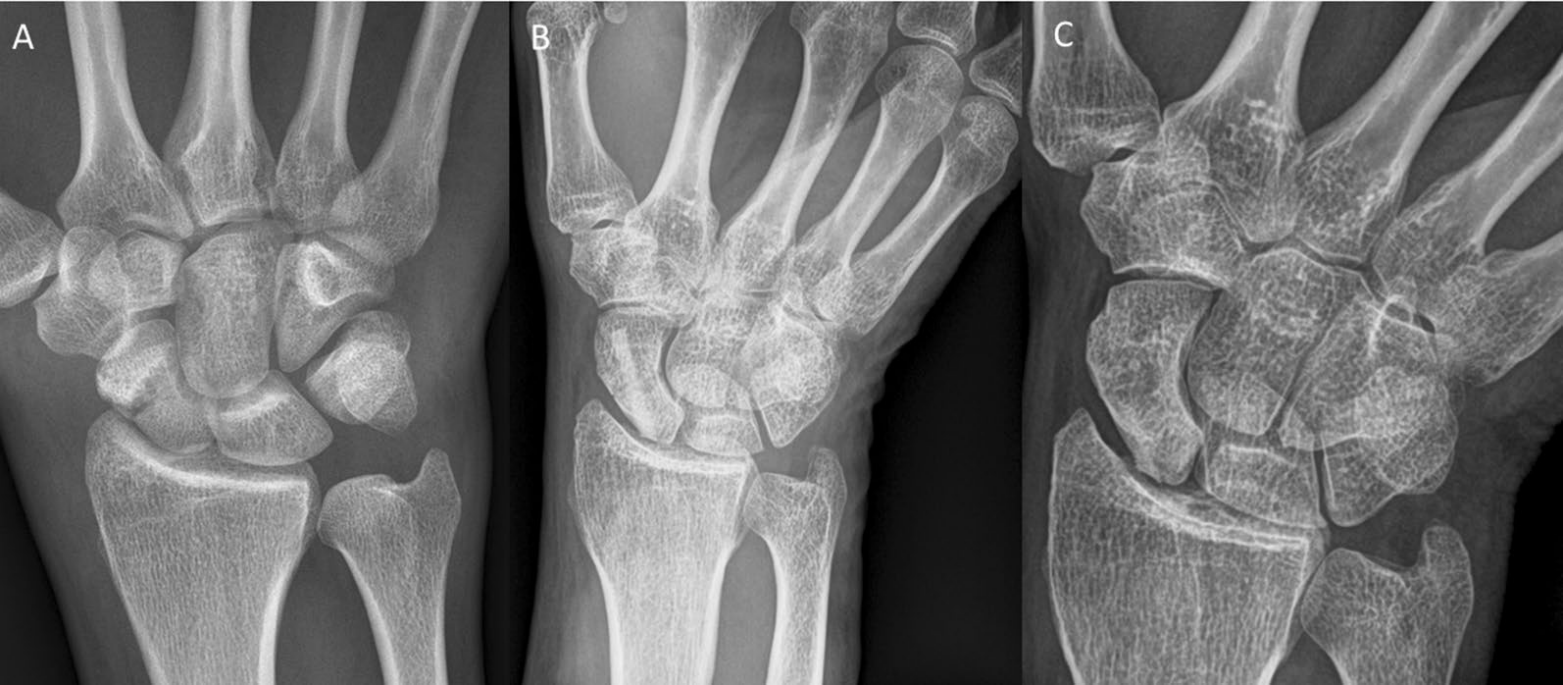
Pseudarthrosis of the scaphoid in the middle third. **A** Pre-surgery. **B** 14 days post-surgery. **C** 27 months post-surgery

**Fig. 5:**
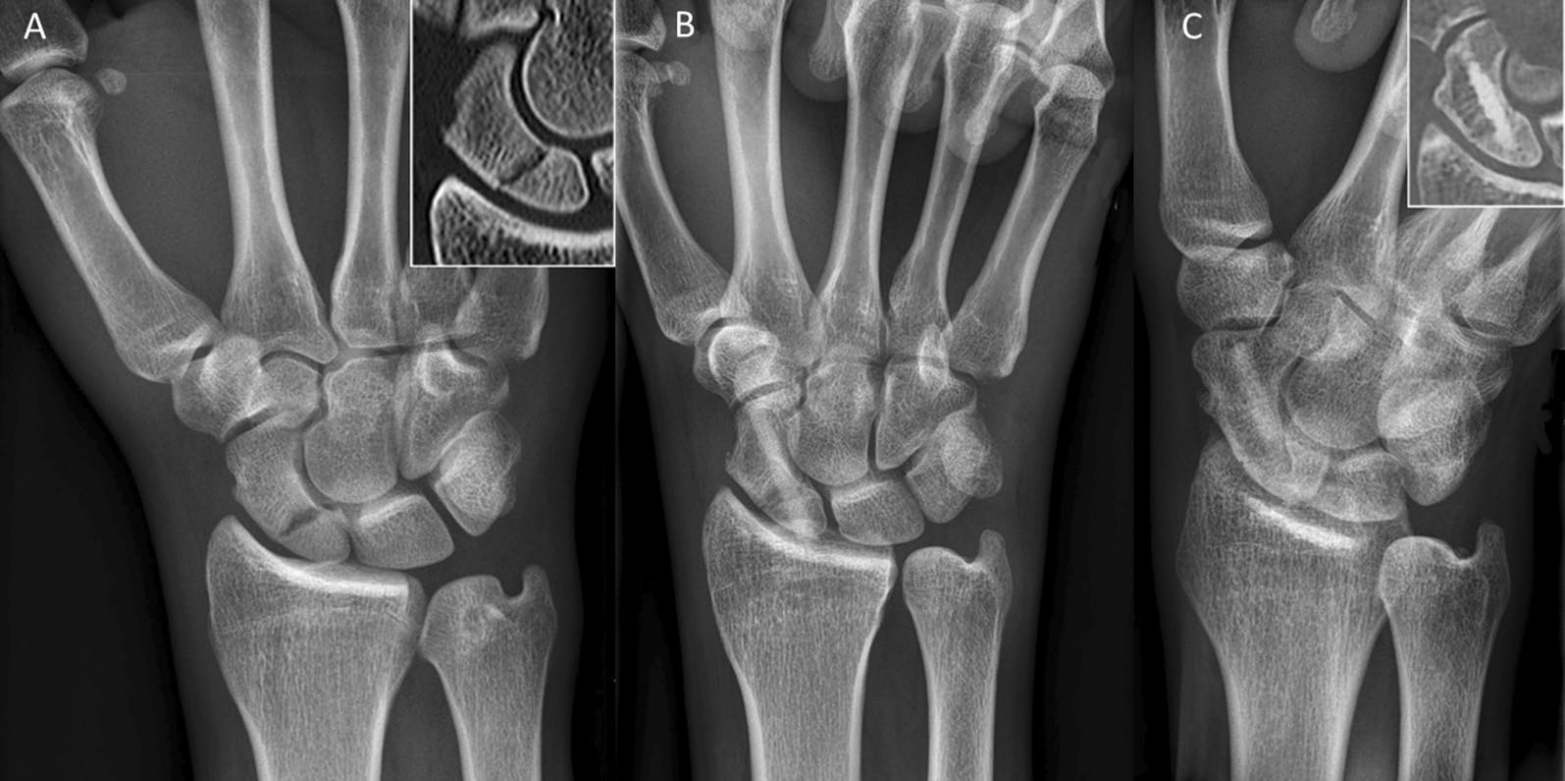
Pseudarthrosis of the scaphoid in the proximal pole. **A** Pre-surgery,* Inset*: CT of the scaphoid. **B** 1 day post-surgery. **C** 10 months post-surgery.* Inset*: CT of the scaphoid

Inclusion criteria were: patients aged 18–99 years; the use of the human allogeneic cortical bone screw (Shark Screw®, Surgebright GmbH, Lichtenberg, Austria; Fig. 1A) for fixation; and at least two follow-ups, at least one of which occurred after 6 months. 75 patients were included in this retrospective study. Since the operating surgeons only used the human allogeneic cortical bone screw, no control group was available for the same time period. Patients were divided into a fresh fracture group (F; 31 patients; Figs. 2 and 3) and 44 pseudarthroses. The pseudarthrosis group was divided into delayed union (DU; time to surgery 3 weeks to 6 months, 16 patients; Figs. 4 and 5) and pseudarthroses (PS; time to surgery > 6 months, 28 patients). The fracture was at the proximal pole in 19 patients (Figs. 3 and 5), in the middle in 55 patients (Figs. 2 and 4), and distal in 1 patient. Classification of the fractures was performed after Krimmer, Szabo and Filan [33–35] to provide a detailed and standardized classification for all surgeons involved. Before surgery, all patients had a CT scan to better see the fracture/pseudarthrosis.

Age, gender, time to surgery, fracture classification after Krimmer, Szabo and Filan [33–35], presence of cysts at the time of surgery, associated injuries, size and number of cortical bone screws used, use of autologous cancellous bone (in addition to the cortical bone screw), palmar or dorsal approach during surgery, exposure of pseudarthroses, bone quality (very good, good, moderate and bad), additional procedures, duration of hospital stay, number and duration (days) of immobilizations, complications, follow-up, cystic brightening, union, nonunion and cortical bone screw fractures, time to union, reoperation and pseudarthroses after surgery were recorded (Tables 1, 2 and 3). Radiographic examination was performed before surgery (posteroanterior views in the neutral wrist position and lateral and special scaphoid views) and at each follow-up. Postoperative X-ray was used to define union. CT scans were only performed in the case of doubt. Routine follow-ups were performed after 6 weeks, 3 months, 6 months and 1 year.

**Table 1:** Patient data

	Fresh	Delayed union	Pseudarthrosis
Patients (*n*)	31	16	28
Age (years)	32* (18–59)	37 (20–70)	28 (18–51)
Sex (male/female)	21/10	13/3	25/3
Right/left side	12/19	8/8	19/9
Time to surgery (days)	7 (0–21)	67^#^ (23–161)	518^§,$^ (178–3940)
Proximal/middle/distal	6/25/0	5/11/0	8/19/1
A2	5	–	1
B1	7	–	–
B2	13	5	5
B3	2	1	4
B4	4	–	-
C	–	8	2
D1	–	1	4
D2	–	1	9
D3	–	–	3
Pseudarthrosis with cyst	0	0	17
Associated injury	12	1	5

*Data are given as median (range); ^#^*p* = 0.000975 for fresh vs. delayed union; ^§^*p* = 0.01076 for delayed union vs. pseudarthrosis; ^$^*p* < 0.0001 for fresh fractures vs. pseudarthrosis

**Table 2:** Surgery data and rehabilitation

	Fresh	Delayed union	Pseudarthrosis
Patients (*n*)	31	16	28
Shark Screw® used			
3.5	25	10	6
4.0	6	6	18
4.5	0	0	4
Autologous spongiosa used	0	1	11
Dorsal/palmar	9/22	12/4*	16/12^#^
Pseudarthrosis exposure	0	0	11
Bone quality			
Very good	18	13	14
Good	12	2	11
Moderate	1	1	3
Additional procedures	6	5	2
Duration of hospital stay (days)	1 (1–21)	1 (0–4)	1 (0–28)
Postoperative immobilization (n)	22	15	26
Duration of fixation (days)	42 (4–90)	28 (14–70)	42 (14–112)
Complications	1	0	0

Data are given as median (range); *p = 0.00476 for fresh vs. delayed union; ^#^*p* = 0.0373 for fresh vs. pseudarthrosis, Fishers exact test

**Table 3:** Post-surgery data

	Fresh	Delayed union	Pseudarthrosis
Patients (*n*)	31	16	28
Follow-up, days (range)	352 (173–1088)	287 (182–853)	326 (187–966)
Cystic brightening	1	0	5
Union (*n*, %)	29 (94)	15 (94)	27 (96)
Nonunion (*n*, %)	2 (6)	1 (6)	1 (4)
Time to union* (days)	115 (24–481)	201 (28–459)	127 (28–966)
Percent union after* (only patients with union were included)			
100 days	38%	13%	37%
200 days	76%	40%	70%
300 days	90%	67%	77%
500 days	100%	100%	93%
Re-operation	4	1	1
Related to bone screw	2	1	0
Pseudarthrosis at last follow-up	1	0	1

Data are given as median (range); *due to the pandemic situation, patients omitted scheduled visits and returned only later for the control; thus, the time to union is longer than it would be under normal conditions

### Surgical technique and postoperative outcome

The decision regarding whether the surgical procedure was performed from the palmar or dorsal site depended on the fracture location and on the preferences of the six individual surgeons. Proximal scaphoid fractures were exclusively operated from the dorsal site. In the case of a humpback deformity or big cysts, an open approach was performed and a corticocancellous autograft was used. In the case of nondisplaced nonunions, all pseudarthroses were refreshed and debrided. The Shark Screw® is a set screw but not a compression screw. Thus, the parts of the fracture are pressed together (with hooks in an open approach or with K-wires when closed reduction is used), and the screw is put in place.

#### Palmar access

A short (approx. 0.5 cm) skin incision was made above the scaphotrapezial joint at the radial border of the tendon of the flexor carpi radialis muscle. After the skin incision, a blunt probe was made with scissors and spread. Under X-ray image monitoring, the K-wire was inserted from the palmar-radial aspect of the scaphoid via the scaphoid tubercle towards the proximal pole of the scaphoid. The intraoperative X-ray image monitoring included at least three projection planes (dorsopalmar, lateral, engraver) and fluoroscopy during forearm rotation. After attaining the correct positioning, the core hole was drilled over the 1.2-mm K-wire. The thickness of the human allogeneic cortical bone screw was determined based on the size of the bone. After drilling the core hole, the thread for the cortical bone screw was cut over the 1.2-mm K-wire. The desired drilling and thread depth were made visible with lateral laser markings. The finished tunnel was rinsed with physiological saline solution to clean out fine bone chips. The cortical bone screw cut was then screwed in, without much resistance, with a hexagonal screwdriver, also under intraoperative X-ray monitoring (Fig. 1B). The skin incision was pulled proximally with a narrow-wound hook and, at the same time, the wrist was bent (Fig. 1C) so that the protruding material of the cortical bone screw could be sawn off just above the bone surface with a very narrow oscillating saw.

#### Dorsal access

A longitudinal incision was performed ulnar in the immediate vicinity of the tuberculum lister. Underneath, the retinaculum extensorum and the wrist capsule were opened while protecting the scapholunar ligament. The surgeon’s thumb and index finger encompassed the thumb saddle joint. The wrist was flexed and 1.2 K-wire was drilled over the proximal scaphoid pole, aiming the K-wire towards the center of the thumb saddle joint or thumb digit. Intraoperative X-ray was then performed, including at least three projection planes (dorsopalmar, lateral, pricker) and fluoroscopy during forearm rotation. Then, as described above, after removing protruding material, the screw was ground back to below the cartilage surface with a small ball milling machine.

As a rule, for postoperative care, immobilization in a cast was required for at least 2 weeks. In most cases, the resumption of extensive physical exertion should only be permitted after 12 weeks.

### Statistical methods

Data are presented as median (range). Due to the non-Gaussian distribution of the data, non-parametric Kruskal–Wallis ANOVA was used for calculating significant differences in quantitative values. Ordinal values were evaluated with contingency tables using Fisher’s exact test for significance. Kaplan–Meier curves were used to show differences in time to union between the groups. A *p* value of < 0.05 was defined as representing a significant difference.

## Results

**Fig. 6:**
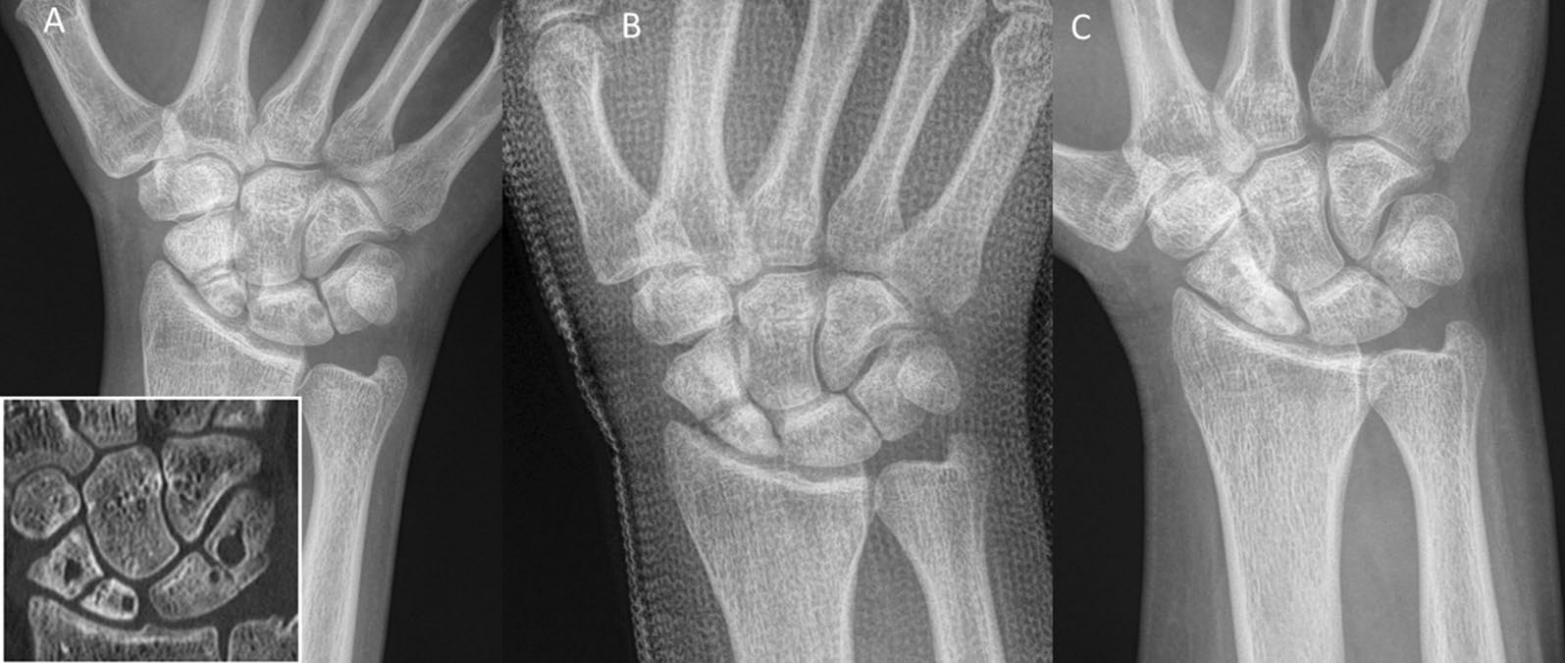
Example of cysts pre-surgery. Pseudarthrosis patient with a fracture in the middle third. **A** Pre-surgery.* Inset*: CT with good visibility of the cysts. **B** 1-day post-surgery. **C** 17 months post-surgery; no cysts are visible

Age and gender were not different between the groups (Table 1). Time to surgery was significantly different between the investigated groups. For fresh scaphoid fractures, the classification [33–35] was A and B, whereas fractures classified as B, C and D were present in delayed-union and pseudarthrosis patients. Seventeen of the 43 pseudarthrosis patients presented with cysts (Fig. 6). These were not detectable post-surgery in all but one case, where the cysts got smaller. Associated injuries were recorded mainly in fresh facture patients; some of them were additional fractures of other bones of the same hand or the opposite hand or hand joint, forearm fractures, rib fractures, knee injuries, foot injuries, and polytrauma (Table 1).

The size of the human allogeneic cortical bone screw used was mainly 3.5 and 4.0 mm (Table 2). All patients with proximal fractures were operated on from the dorsal site, whereas 67% of the patients with a fracture in the middle were operated from the palmar site. Post-operative immobilization was indicated in 93% of the pseudarthrosis patients and in 71% of patients with fresh fractures. Eleven of the 12 patients who did not receive immobilization had fractures in the middle third of the scaphoid. The decision to omit immobilization depended on the location of the scaphoid fracture, the diameter of the cortical bone screw used, the time between fracture and surgery, and the experience of the surgeon. Complications (complex regional pain syndrome) were noticed in 1 patient (Table 2).

Mean follow-up was 13 months (6 months to 3 years, Table 3). The union rate was 95–96%. Re-operations related to the cortical bone screw were performed in 3 patients (4%); they were related to nonunion in 2 patients, and in 1 patient the cortical bone screw was too long and had to be shortened.

There were 31 fresh scaphoid fractures. The healing process of fresh scaphoid fractures after fixation with the human allogeneic cortical bone screw is shown in Fig. 2 (middle third) and Fig. 3 (proximal pole). Median time to union was 115 days (16 weeks) for the fresh fracture group. There were two nonunions in the fresh fracture group (Table 3).

**Fig. 7:**
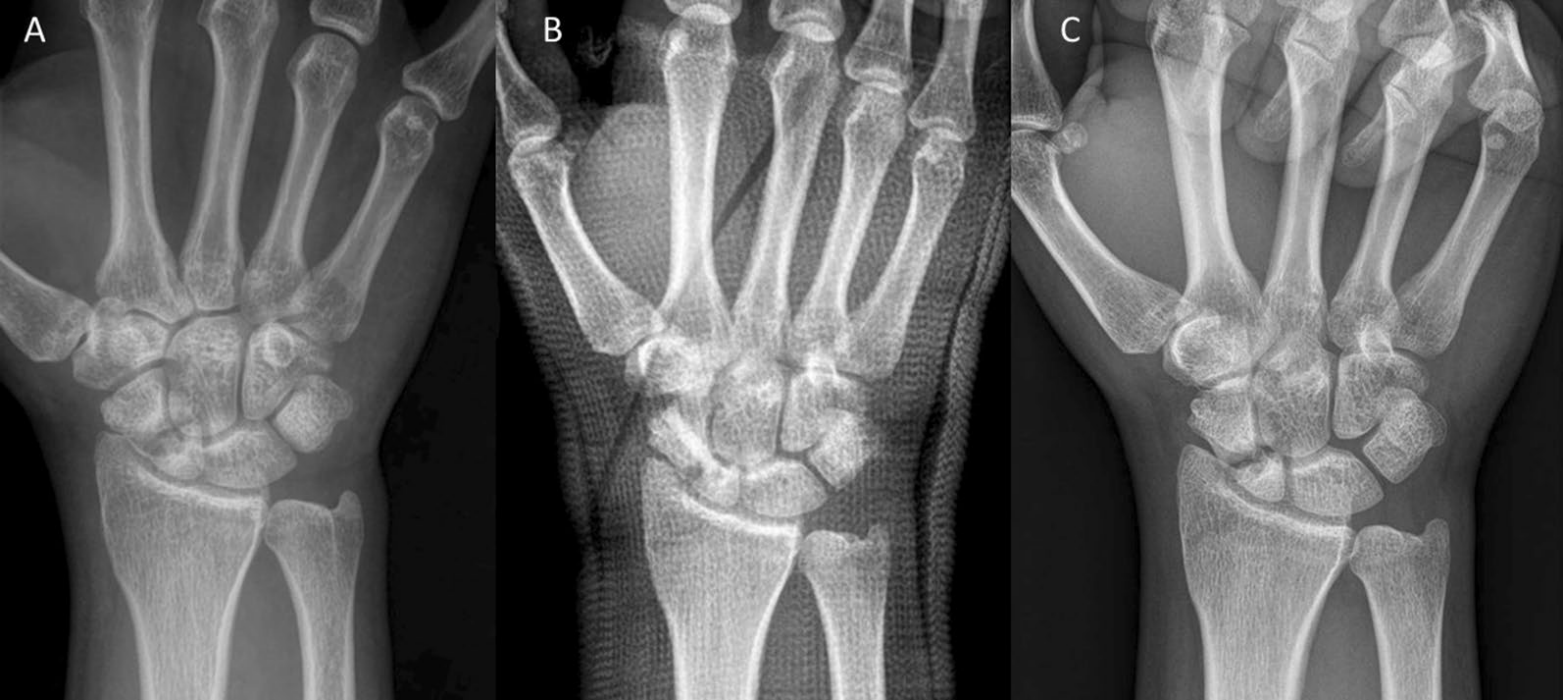
Example of nonunion. The patient had pseudarthrosis of the scaphoid in the middle third. **A** Pre-surgery. **B** 6 weeks post-surgery. The screw was not placed deep enough into the proximal pole. **C** 16 months post-surgery. The screw was fully integrated in the bone, but union was not achieved. Re-operation is scheduled

Twelve patients in the pseudarthrosis group needed autologous spongiosa from the distal radius or from the pelvis (Table 2). Pseudarthrosis exposure had to be performed due to the dislocation of the fracture or due to the deformity of the wrist in 11 patients. Median time to union was 127 days (18 weeks) for the pseudarthrosis group and 201 days (28 weeks) for the delayed union group (Table 3). After 100 days, union was observed in 38% of the fresh fracture patients, 13% of the delayed union patients and 37% of the pseudarthrosis patients. These percentages increased to 76%, 40% and 70% after 200 days and to 90%, 67% and 77% after 300 days, respectively. Bone healing in pseudarthrosis patients is shown in Fig. 4 (middle third) and Fig. 5 (proximal pole). Complete remodeling of the cortical bone screw without a scar was achieved 27 months after surgery (Fig. 4C). In the delayed union group and the pseudarthrosis group, only one nonunion at the proximal pole was detected in each group. Non-compliance of a heavy-smoking patient who performed heavy manual work caused one nonunion (delayed union group). In one pseudarthrosis patient (Fig. 7), the Shark Screw® was not placed deep enough into the proximal pole. The bridging of the fracture gap was incomplete.

**Fig. 8:**
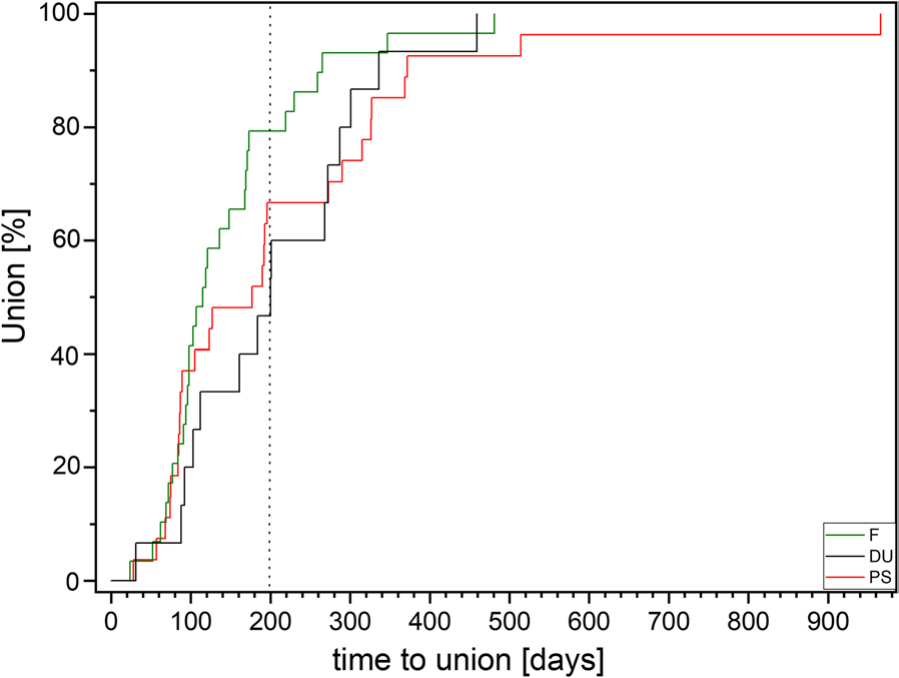
Kaplan–Meier curve for time to union: only patients with unions are included. Patients are divided into those with a fresh fracture (time between injury and surgery was < 3 weeks, F), delayed union (time between injury and surgery was between 3 weeks and 6 months, DU) and pseudarthrosis (time between injury and surgery was > 6 months, PS). The* vertical dotted line* is included to facilitate reading the figure at 200 days

Plotting time to union in a Kaplan–Meier curve, no significant differences were observed between the fresh fracture group, the delayed union group and the pseudarthrosis group (Fig. 8).

## Discussion

The most important finding of this retrospective study is that the human allogeneic cortical bone screw is totally transformed into the host bone after several months [12]. This allows us to conclude that after this time, the scaphoid looks like as never broken before (Fig. 4C) and as also as shown by recent publications [9–12]. Three of the four nonunions in our retrospective study were proximal pole fractures; excluding these, as done in the SWIFFT study [14], would have improved our union rate result to 98.7%. The results are in agreement with findings by Jaminet et al. [20], who described nonunion rates of 3% for the middle third and 18% for the proximal pole in pseudarthrosis patients. Others describe 14% [32] or 30% [22] nonunion when treating the proximal pole in pseudarthrosis patients. For pseudarthrosis patients in our patient cohort, the union rate was 96%, in agreement with results reported in the literature: 84–90% when using vascularized grafts [17, 36] and between 80% and 87% when using non-vascularized grafts. Hegazy et al. [37] described union rates of 90% and 94% for corticocancellous and cancellous-bone-only autografts, respectively, in pseudarthrosis patients.

Additionally, this retrospective study demonstrated a high union rate (94–96%), especially in pseudarthrosis patients (96%), when using the human allogeneic cortical bone screw for fixation. The two nonunions (4%) in the pseudarthrosis patients were due to non-compliance and a fracture gap bridging that was too short (Fig. 7). Median time to union for the pseudarthrosis group was 18 weeks, as described by Pinder et al. [17]. Mean time to union was prolonged in the presented study due to the coronavirus pandemic, as patients omitted follow-ups and union could only be recorded after the pandemic restrictions were canceled, at which point it was far beyond the normal time to union. Another factor for the long time to union could be the high number of patients with a classification of D2 or higher (17%), who show a longer time to union. This is in agreement with Higgins et al. [38], who described fracture location, duration since injury, previous nonunion, smoking and fracture characteristics (other than location) as the main preoperative factors which impact the success of scaphoid nonunion surgery.

Merrell et al. [28] described lower (47%) union rates with a non-vascularized wedge graft. Aibinder et al. [30] observed a 71% union rate for structural iliac crest [30]. Using autologous bone, taken from the tibia of the patient and shaped intraoperatively, to fix the scaphoid fracture with an autologous bone screw yielded a union rate of 80% with a high complication rate and long surgery time [6].

In the presented study, we recorded only one complication in 75 patients (1.3%, complex regional pain syndrome) and arthrosis at the last follow-up in 2 patients where union could not be achieved. This favorable outcome is related to the use of the human allogeneic cortical bone screw, which avoids donor site morbidity and problems related to metal hardware. Complication rates were reported to be between 21 and 26%, depending on the graft used [23, 37, 39]. The meta-analysis by Feeley et al. [2] regarding the use of bioabsorbable fixation methods revealed 23% complications, and the SWIFFT study reports 14% in the surgical group [14]. Reigstad et al. [24] described increased arthrosis around the implanted screw.

The union rate in the fresh fracture group (94%) was similar to values described by Dias et al. [14], even though they included only non-displaced or minimally displaced patients and excluded patients with proximal pole fractions. Median time to union for a fresh fracture group as described by Pinder et al. [17] was 16 weeks, with 18 weeks and 4 months described by Tada et al. [40], who mainly investigated patients who had their surgery between 6 weeks and 6 months after the injury. The percentage of unions in the fresh fracture group after 100 days (14 weeks) was 38% and was similar to results presented by the SWIFFT study after 12 weeks (47% achieved full union, 68% achieved almost full union [14]).

Brcic et al. [8] describes the human allogeneic cortical bone screw as a sterilized cortical bone allograft for osteosynthesis. Furthermore, they describe that the human allogeneic cortical bone screw has a fine thread with high rotational stability and accuracy of fit, and it offers the ideal conditions for the migration, proliferation and differentiation of all bone cells [8]. One aspect to consider with every application of the Shark Screw® is that bone grafting means bone reconstruction. The original bone is reconstructed. This cortical homoplasty stimulates the environment for bone renewal. The osteogenic response is clearly visible around the Shark Screw® in the CT. This is particularly important in the case of pseudarthrosis when bone defects with bone loss (cysts, necrosis) have occurred. In order to fill these bone defects with cortical bone in the best feasible way (cortical homoplasty), the largest possible diameter of the Shark Screw® should always be selected, even if the biomechanical conditions would allow a thinner screw. This allograft is remodeled without scars by the physiological bone metabolism and, on average, within a year, it is converted into the patient's own bone [8, 10, 12] (Fig. 4C).

Time between injury and surgery was reported as 7–50 (mean 14.8) weeks [40], similar to the median for our patient cohort (same day to 63 weeks).

Donor site morbidity, as described in the literature [6, 19, 41], can be avoided by using the human allogeneic cortical bone screw. Distal radius bone graft donor site complications are underreported or may be attributed to the recipient site because of their close proximity [17]. In our experience, and in agreement with the literature [6], it takes about an extra 30–45 min to prepare autologous cortical bone.

Hardware removal is another problem which is avoided by using the human allogeneic cortical bone screw. In 17% [41] to 67% [42] of the patients, the hardware had to be removed. Keller et al. [23] reported implant irritation which healed only after implant removal. Biomaterials are one alternative, but these materials and their degradation products may interfere with bone healing [2]. 21,000 hardware removals were performed in Austria in 2016 (personal communication, Gebietskrankenkasse Oberösterreich). Each hardware removal costs 2350.62 € (2016), the patient stays on average 3.5 days in the hospital, and 1 hospital day costs 923 € (2019). In Upper Austria, sick leave is a minimum of 15 days after hardware removal (and can be up to 35 days), with a daily income of 140 € (source of all data: personal communication, Gebietskrankenkasse Oberösterreich). In total, this comes to 7681.12 € per hardware removal. Estimating that 1% of the hardware removals in Austria were due to hardware removal after scaphoid fixation, this accumulates to 1,613,035.20 € per year, which could be saved by using the human allogeneic cortical bone screw. These are only the costs of hardware removal and sick leave; they do not include the time saved in the operation theater at the initial operation and other costs, such as those of additional visits and medication. In a very recent publication, Andersson et al. [15] described that the frequency of settled claims of scaphoid fractures was 20% higher than for general complaints [15]. Furthermore, they found that 56% of the claims were due to delayed diagnosis and 41% were due to misdiagnosis [15]. In 60% of the patients with a settled claim, it resulted in medical invalidity [15]. They further described that total costs of 15,700 € per patient (indirect and direct costs) were calculated for misdiagnosed and mistreated patients [15].

Langegger [43] describes a future perspectives on the use of the human allogeneic cortical bone screw, Pastl et al. [9] reported the first results obtained with the cortical bone screw in hand and foot surgery, and Amann et al. [10] and Hanslik-Schnabel et al. [12] described results of using the screw in foot surgery and Huber et al. [11] in shoulder surgery.

There are several limitations of this study. First, the retrospective nature of the study. Second, due to the explorative nature of the study and ethical concerns, no control group, using a metal screw, instead of the cortical bone screw was available for comparison. Third, no scores were available to interpret the results. Further prospective studies are needed to confirm these preliminary findings.

In conclusion, using the human allogeneic cortical bone screw (Shark Screw®) led to similar union rates to those presented in the literature for other scaphoid fracture fixation techniques and enabled a short and low-invasive procedure without any donor site morbidity and without the necessity to remove the hardware in a second surgery. The pseudarthrosis patient group received a particularly strong benefit from this new procedure. The physiological bone metabolism remodels the cortical bone screw without scars.

## Data Availability

Due to the sensitivity of the raw data, all data are available from the first author on request.
